# The Anti-Inflammatory Effect of KS23, A Novel Peptide Derived From Globular Adiponectin, on Endotoxin-Induced Uveitis in Rats

**DOI:** 10.3389/fphar.2020.585446

**Published:** 2021-01-12

**Authors:** Xin Shi, Shaopin Zhu, Huiyi Jin, Junwei Fang, Xindan Xing, Yihan Wang, Hanying Wang, Chingyi Wang, Tian Niu, Kun Liu

**Affiliations:** ^1^ Department of Ophthalmology, Shanghai General Hospital, Shanghai Jiao Tong University School of Medicine, Shanghai, China; ^2^ National Clinical Research Center for Eye Diseases, Shanghai, China; ^3^ Shanghai Key Laboratory of Ocular Fundus Diseases, Shanghai, China; ^4^ Shanghai Engineering Center for Visual Science and Photo Medicine, Shanghai, China; ^5^ Shanghai Engineering Center for Precise Diagnosis and Treatment of Eye Diseases, Shanghai, China

**Keywords:** globular adiponectin, endotoxin-induced uveitis, inflammation, nuclear factor kappa B, lipopolysaccharide

## Abstract

**Purpose:** Adiponectin has been shown to exert potent anti-inflammatory activities in a range of systemic inflammatory diseases. This study aimed to investigate the potential therapeutic effects of KS23, a globular adiponectin-derived peptide, on endotoxin-induced uveitis (EIU) in rats and lipopolysaccharide (LPS)-stimulated mouse macrophage-like RAW 264.7 cells.

**Methods:** EIU was induced in Lewis rats by subcutaneous injection of LPS into a single footpad. KS23 or phosphate-buffered saline (PBS) was administered immediately after LPS induction via intravitreal injection. Twenty-four hours later, clinical and histopathological scores were evaluated, and the aqueous humor (AqH) was collected to determine the infiltrating cells, protein concentration, and levels of inflammatory cytokines. *In vitro*, cultured RAW 264.7 cells were stimulated with LPS in the presence or absence of KS23, inflammatory cytokine levels in the supernatant, nuclear translocation of nuclear factor kappa B (NF-κB) subunit p65, and the expression of NF-kB signaling pathway components were analyzed.

**Results:** KS23 treatment significantly ameliorated the clinical and histopathological scores of EIU rats and reduced the levels of infiltration cells, protein, tumor necrosis factor-α (TNF-α) and interleukin-6 (IL-6) in the aqueous humor. Consistently, KS23 decreased the expression of TNF-α and IL-6 in the supernatant of LPS-stimulated RAW 264.7 cells and inhibited the LPS-induced nuclear translocation of NF-κB p65 and the phosphorylation of IKKα/β/IκBα/NF-κB.

**Conclusion:** The *in vivo* and *in vitro* results demonstrated the anti-inflammatory effects of the peptide KS23 and suggested that KS23 is a compelling, novel therapeutic candidate for the treatment of ocular inflammation.

## Introduction

Uveitis is a group of intraocular inflammatory diseases involving the uvea, retina, retinal vessels, and vitreous, and it is one of the leading causes of vision loss, accounting for 10–20% of legal blindness every year (Chang and Wakefield, 2002; Durrani et al., 2004; Nguyen et al., 2011). Uveitis has diverse causes, including bacterial and viral infections, as well as noninfectious causes, such as Fuchs heterochromic iridocyclitis and HLA-B27-associated uveitis (de Smet et al., 2011). Corticosteroids are recommended as the first-line therapy for uveitis (Jabs et al., 2005); however, long-term local or systemic use may result in various adverse effects, such as crystalline lens opacity, ocular hypertension, and abnormal glucose and lipid metabolism (Kok et al., 2005; Pavesio and Decory, 2008; Siddique et al., 2011). Therefore, safe and effective treatment strategies are needed.

Endotoxin-induced uveitis (EIU) is an experimental model of acute ocular inflammation induced by the topical application of lipopolysaccharide (LPS), which is a constituent of Gram-negative bacterial cell walls, into footpads of rodents (Rosenbaum et al., 1980). This model induces an acute, predominately anterior, uveitis that peaks approximately 24 h after injection (Chang et al., 2008). EIU has been widely accepted as an *in vivo* model for assessing the pharmacological and immunological effects of drugs on intraocular inflammation (Uchida et al., 2017; Shoeb et al., 2018).

Adiponectin (APN) is a 244-amino acid long polypeptide chain secreted mainly by adipocytes and released into circulation (Yamauchi et al., 2003). APN consists of an N-terminal collagen region and a C-terminal globular adiponectin (gAPN) region, which has been shown to be a key component of the biological activity of adiponectin (Fruebis et al., 2001). The biological actions of APN are mediated through its seven-transmembrane receptors, adipoR1 and adipoR2 (Yamauchi et al., 2003). APN was originally reported to play a critical role in lipid metabolism and insulin sensitization (Ouchi et al., 2011); however, accumulating evidence suggests that APN also exerts significant anti-inflammatory properties and immunomodulatory effects (Aprahamian et al., 2009; Bobbert et al., 2011; Piccio et al., 2013). APN inhibits the activation of pro-inflammatory transcription factors IL-6, TNF-α, and interferon (IFN)-γ, and promotes the expression of anti-inflammatory genes such as IL-10 (interleukin-10) and heme oxygenase-1 (HO-1) (Wulster-Radcliffe et al., 2004; Ajuwon and Spurlock, 2005; Mandal et al., 2010). Moreover, APN suppresses immune cell activation and adhesion to target cells (Ouchi et al., 2000; Ohashi et al., 2010). APN-deficient mice exhibited severe psoriasiform inflammation in a mouse model of psoriasiform dermatitis, and exogenous adiponectin rescued exacerbated dermatitis (Shibata et al., 2015).

The therapeutic application of APN has been hindered by its complex quaternary structure and rapid turnover *in vivo* (Halberg et al., 2009; Holland and Scherer, 2013). Previously, we developed the novel peptide KS23 with 23 amino acids (KDKAMLFTYDQYQENNVDQASGS RNPRGEEGGPW), derived from gAPN and demonstrated its beneficial effect in experimental autoimmune uveitis (EAU) (Niu et al., 2019). Since the clinical, biochemical, immunological and histological characteristics of EIU (acute anterior uveitis) differ from those of EAU (chronic posterior uveitis) (Yadav et al., 2011; Gutowski et al., 2017), it remains unclear whether KS23 has a protective effect in EIU. Therefore, in the present study, we evaluated the anti-inflammatory properties of KS23 in the rat EIU model, as well as an *in vitro* model, the LPS-stimulated mouse macrophage cell line RAW 264.7, to elucidate the underlying mechanisms. Our results showed that KS23 prevented ocular inflammatory changes in rats associated with EIU and suppressed the IKKα/β/IκBα/NF-κB signaling pathway in LPS-stimulated RAW 264.7 cells. These findings demonstrate that KS23 could be a potential therapeutic agent for ocular inflammation.

## Materials and Methods

### Peptide Preparation

The peptide KS23 (KDKAMLFTYDQYQENNVDQASGS), which was derived from one conserved amino acid sequence of gAPN, and the negative control scrambled peptide TQ23 (TADYNGMKANVDQQYSESKDFLQ) from the same domain were synthesized by ChinaPeptides Co., Ltd. (Shanghai, China) through solid phase, high-performance liquid chromatography. The peptides had a purity >95%, were detected by electrospray ionization mass spectrometry, and were subpacked and finally lyophilized into powder. The peptides were subsequently stored at −20 °C.

### Animals

Eight-week-old male Lewis rats (160–220 g body weight) were obtained from the Shanghai Laboratory Animal Center of the Chinese Academy of Sciences and maintained in standard animal cages. All animal experiments were performed in accordance with the ARVO Statement for the use of Animals in Ophthalmic and Vision Research and adhered to the guidelines required by the Ethics Committee of Shanghai General Hospital, Shanghai Jiao Tong University, School of Medicine, China.

### Endotoxin-Induced Uveitis

Rats were anesthetized by intraperitoneal injection of pentobarbital (5.47 g/100 ml saline). The EIU rat model was established via subcutaneous injection into a single footpad of 100 µL of sterile saline containing 200 mg LPS from *Escherichia coli*, o55:B5 (Sigma-Aldrich, St. Louis, MO, United States). For the control group, each rat was injected with the same volume of saline. Immediately after LPS injection, the rats were randomly injected intravitreally with 10 μL of phosphate-buffered saline (PBS, pH 7.4), KS23 (1, 5 and 10 µg/µL), TQ23 (10 µg/µL), or dexamethasone (DXM) (10 µg/µL) (Sigma-Aldrich) diluted in PBS.

Before injection, rats were anesthetized via intraperitoneal injections with ketamine (72 mg/kg body weight) and xylazine (4 mg/kg body weight). The pupils were dilated using 0.5% phenylephrine hydrochloride and 0.5% tropicamide (Mydrin-P; Santen Pharmaceutical, Osaka, Japan). 0.4% oxybuprocaine hydrochloride (Benoxil; Santen Pharmaceutical) as topical anesthesia were applied. Intravitreal injection was performed with a 33-gauge microsyringe (Hamilton Co., Reno, NV, United States) under a microscope.

All rats were randomly divided into the following seven groups (n = 20 in each group): 1) the control group, rats were administered 100 µL of saline to the footpad, and injected with 10 µL of PBS intravitreally; 2) EIU + PBS group, EIU rats were injected with 10 µL of PBS intravitreally; 3) EIU + TQ23 group, EIU rats were injected with 10 µg/µL TQ23 intravitreally; 4–6) EIU + KS23 groups, EIU rats were injected with KS23 (1, 5, or 10 µg/µL, separately) into the vitreous cavity; and 7) EIU + DXM group, EIU rats were treated with 10 µg/µL DXM.

### Clinical Scoring of Ocular Inflammation

Clinical scoring was conducted at the 24 h timepoint after modeling and was accomplished by examining the eyes under a microsurgery microscope (Olympus Optical Ltd., Tokyo, Japan). Images were taken with a digital camera (Canon Inc., Tokyo, Japan). The anterior segment of the eye was observed, including conjunctival vasodilation, tortuosity, hyperemia, conjunctival edema, postcorneal sedimentation, anterior chamber flash, fiber exudation, posterior iris adhesion, and pupillary membrane closure. The scores were rated independently according to the Behar-Cohen Rating criteria by two blinded experimenters (Behar-Cohen et al., 1998). The severity of EIU was graded from 0 to 4 as follows: 0, no inflammatory reaction; 1, discrete dilation of the iris and conjunctival vessels; 2, moderate dilation of the iris and conjunctival vessels with moderate flare in the anterior chamber; 3, intense iridial hyperemia with flare in the anterior chamber; and 4, the same clinical signs as 3 plus the presence of fibrinous exudates in the pupillary area and miosis.

### Infiltrating Cells and Proteins in the Aqueous Humor

The aqueous humor (AqH) was collected via anterior chamber puncture of the eye with a 30-G needle 24 h after LPS injection. The sample was diluted 1:5 with Trypan blue solution and then added to the hemocytometer. The number of inflammatory cells in the four large fields of view was manually counted under an inverted phase contrast microscope (200×), and the number of cells per microliter was determined by averaging the results of four fields from each sample. The number of inflammatory cells in the solution was calculated. The total protein concentration in the AqH was measured by a bicinchoninic acid (BCA) protein assay reagent kit (Pierce, Rockford, IL, United States).

### Levels of Tumor Necrosis Factor-α and Interleukin-6 in Aqueous Humor

The AqH from both eyes of each rat was centrifuged at 2,500 rpm for 20 min at 4 °C. The concentrations of TNF-α and IL-6 in the AqH were quantified with the corresponding ELISA kits (R&D Systems, Minneapolis, MN, United States) according to the manufacturer’s instructions.

### Histopathological Examination

The rats were euthanized 24 h after LPS administration, and the eyes were enucleated immediately and fixed in 4% paraformaldehyde for 24 h. Subsequently, the eyes were embedded in paraffin. Serial 5 μm sections were cut starting at the optic nerve head and stained with hematoxylin and eosin (H&E). Infiltrating leukocytes in the anterior chamber were counted under a light microscope, and the severity of inflammation was scored in a blinded manner according to the report by Tilton (Tilton et al., 1994). The severity of EIU was graded from 0 to 4 as follows: 0, normal tissue; 1+, dilated iris vessels and thickened iris stroma with exudate, protein, and/or a few scattered inflammatory cells in the anterior chamber; 2+, infiltration of inflammatory cells into the stroma of the iris and/or ciliary body, with a moderate number of inflammatory cells within the anterior chamber; 3+, heavy infiltration of inflammatory cells within the iris stroma and ciliary body and heavy infiltration of inflammatory cells within the anterior chamber; 4+, heavy exudation of cells in dense protein aggregation in the anterior chamber and inflammatory cell deposits on the corneal endothelium.

### Cell Culture

Mouse macrophage-like RAW 264.7 cells were purchased from the American Type Culture Collection (Manassas, VA, United States), and cultured in Dulbecco’s modified Eagle’s Medium enriched with 10% (vol/vol) fetal bovine serum (FBS), penicillin (100 U/ml), and streptomycin (100 µg/ml, all Thermo Fisher Scientific, Waltham, MA, United States) at 37 °C in a 5% CO_2_ atmosphere.

Primary Human Corneal Epithelial Cells (HCEpiC) were purchased from ScienCell Research Laboratories (San Diego, CA, United States), and cells were cultured according to supplier’s instructions, and cells with passage numbers 5–12 were used in the experiments.

### Cell Viability Assay

RAW 264.7 cells and primary HCEpiC cells were plated in 96-well plates and serum-starved for 24 h. Then, the cells were treated with LPS (100 ng/ml), KS23 (0.1, 1, or 10 µM) and TQ23 (10 µM) separately for 24 h, while the control group was left untreated. Cell viability was assayed using a CellTiter 96 AQueous One Solution Cell Proliferation assay (MTS) kit (Promega, Madison, WI, United States) according to the manufacturer’s instructions.

### Tumor Necrosis Factor-α and Interleukin-6 Levels in Lipopolysaccharide-Stimulated RAW 264.7 Cells

RAW 264.7 cells were seeded and serum-starved for 24 h. Then, the cells were pretreated with KS23 (0.1, 1, or 10 µM, separately) or TQ23 (10 µM) for 30 min and further stimulated with 100 ng/ml LPS for 1 h. After stimulation, the culture supernatant was collected and the levels of cytokines (TNF-α and IL-6) were analyzed using corresponding ELISA kits (R&D Systems) according to the manufacturer’s instructions.

### The mRNA Levels of Tumor Necrosis Factor-α and Interleukin-6 in Lipopolysaccharide-Stimulated RAW 264.7 Cells

The mRNA levels of IL-6 and TNF-α cytokines in RAW 264.7 cells were detected by Quantitative Real-Time PCR (qPCR). After pretreated with KS23 (0.1, 1, or 10 µM, separately) or TQ23 (10 µM) for 30 min and further stimulated with 100 ng/ml LPS for 1 h, the total RNA was extracted with TRIzol (Invitrogen, Carlsbad, CA, United States). Complementary DNA was prepared using PrimeScriptTM RT reagent Kit (TaKaRa Biotechnology, Co., Ltd., Dalian, China) according to the manufacturer’s instructions. The qPCR was performed using an ABI Prism 7500 Sequence Detection System (Applied Biosystems, Foster City, CA, United States), and the values for each gene were normalized to the level of glyceraldehyde 3-phosphate dehydrogenase (GAPDH).

The specific primers used for qPCR were as follows: mouse GAPDH (forward, 5′-TGG​CCT​TCC​GTG​TTC​CTA​C-3′ and reverse, 5′-GAG​TTG​CTG​TTG​AAG​TCG​CA-3′); mouse IL-6 forward, 5′-CTG​CAA​GAG​ACT​TCC​ATC​CAG and reverse, 5′-AGT​GGT​ATA​GAC​AGG​TCT​GTT​GG-3′); and mouse TNF-α (forward, 5′-CTG​AAC​TTC​GGG​GTG​ATC​GG and reverse, 5′-GGC​TTG​TCA​CTC​GAA​TTT​TGA​GA-3′).

### Western Blotting

RAW 264.7 cells were seeded and serum-starved for 24 h. Then, the cells were pretreated with PBS, TQ23 (10 µM), or KS23 (0.1, 1, or 10 µM) for 30 min before being further treated with LPS (100 ng/ml) for 1 h. Whole cell extracts were prepared on ice with lysis buffer (Thermo Fisher Scientific) containing protease and phosphatase inhibitors. The protein concentrations were measured using a BCA assay (Thermo Fisher Scientific). Each sample was loaded onto 10% sodium dodecyl sulfate-polyacrylamide gels, separated by electrophoresis and transferred onto polyvinylidene fluoride (PVDF) membranes (Millipore, Bedford, MA, United States). Then, the PVDF membranes were blocked with 5% nonfat milk for 2 h and incubated overnight at 4 °C with primary antibodies that specifically recognized NF-κB (1:1,000; Cell Signaling Technology, Danvers, MA, #8242), inhibitor of NF-κB (IκBα) (1:1,000; Cell Signaling Technology #4812), IκB kinase (IKK) α/β (1:1,000; Abcam, ab178870, Cambridge, United Kingdom), phospho-NF-κB^Ser536^ (1:1,000; Cell Signaling Technology, #13346), phospho-IκBα^Ser32^ (1:1,000; Cell Signaling Technology, #2859), phospho-IKKα/β^Ser176/180^ (1:1,000; Cell Signaling Technology, #2697), adiponectin receptor 1 (AdipoR1) (1:1,000; Abcam, ab126611), or β-actin (1:1,000; Cell Signaling Technology, #3700). Next, the PVDF membranes were incubated with the corresponding horseradish peroxidase-conjugated secondary antibodies (1:10,000; Cell Signaling Technology, #7076, #7074) for 1 h at room temperature. The protein band signals were detected with an ECL system detection system (Pierce, Rockford, IL, United States), and the band density was determined using ImageJ software (National Institutes of Health, United States).

### Immunofluorescence Staining for Nuclear Factor-κB

After serum starvation for 24 h, RAW 264.7 cells were seeded on coverslips in 24-well plates pretreated with KS23 (10 µM), TQ23 (10 µM) or PBS for 30 min and then further stimulated with 100 ng/ml LPS for 1 h. The control group was left untreated. After stimulation, the cells were washed with PBS, fixed with 4% paraformaldehyde for 15 min and permeabilized with 0.1% Triton X-100 for 10 min at room temperature. The cells were blocked with 5% goat serum for 1 h at room temperature and then incubated overnight at 4 °C with a rabbit anti-NF-κB p65 antibody (1:200; Cell Signaling Technology, #8242). The coverslips were then washed three times with PBS and incubated with an Alexa 488-conjugated anti-rabbit secondary IgG (1:500; Thermo Fisher Scientific, A-11008) for 1 h at room temperature. The cells were counterstained with DAPI (Sigma-Aldrich) for 5 min after being washed three times with PBS. The coverslips were visualized and photographed under a confocal fluorescence microscope (LSM 510, Carl Zeiss, Gottingen Germany). All coverslips were photographed under the same exposure conditions. The number of cells with nuclear translocation was counted and the percentage of translocated cells compared to the total number of cells was calculated.

### Statistical Analysis

All data are presented as the means ± SD. Statistical analyses were performed using one-way analysis of variance (ANOVA) with Bonferroni’s correction using Prism 6.0 (GraphPad Software, San Diego, CA, United States). A value of *p* less than 0.05 was considered statistically significant.

## Results

### Effect of KS23 on the Clinical Inflammation Score in the Anterior Chamber

**FIGURE 1:**
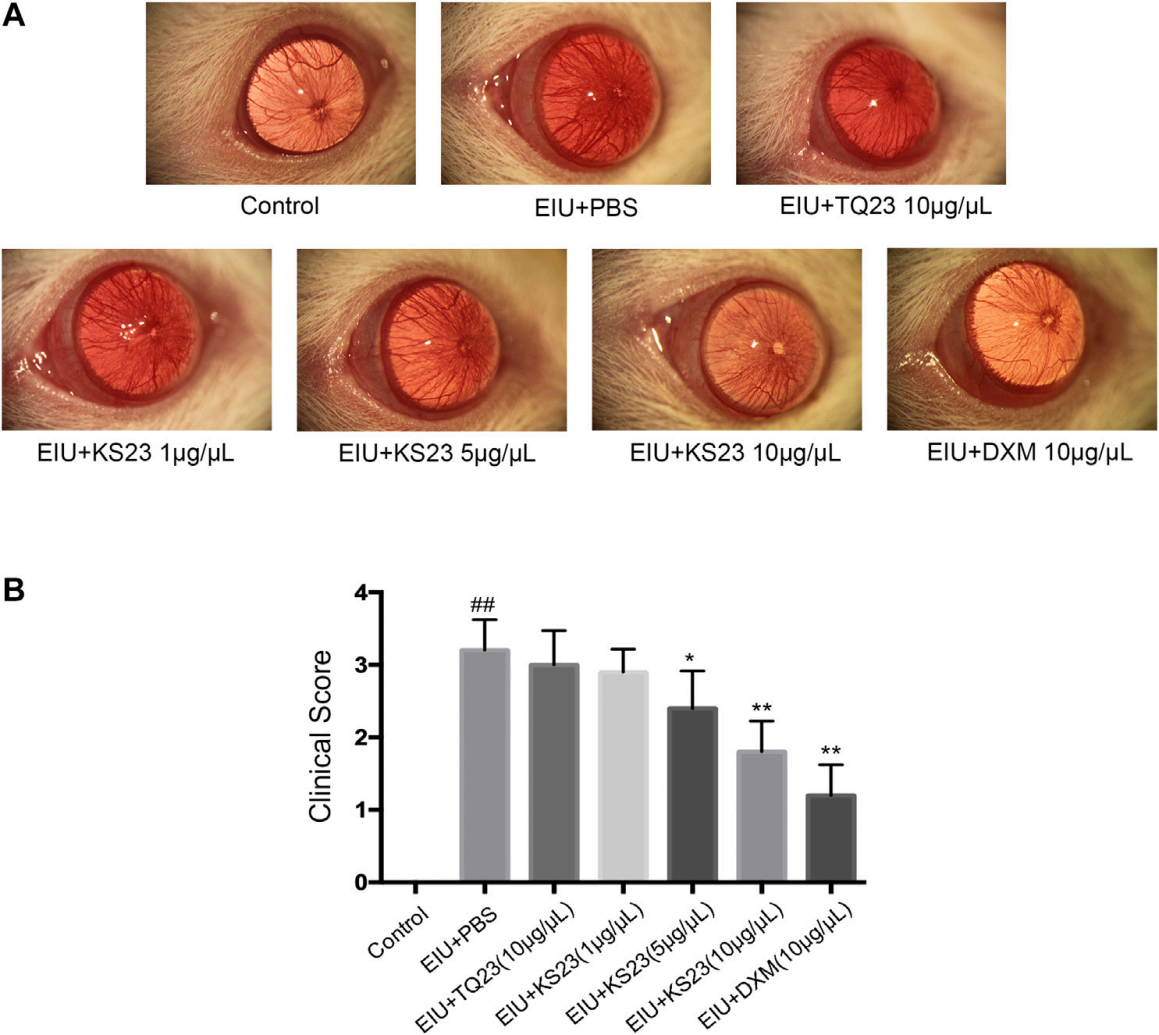
The anti-inflammatory effect of KS23 on the clinical score of the anterior segment of EIU rats. Immediately after LPS injection, the rats were randomly injected intravitreally in both eyes with PBS, TQ23 (10 µg/µL), KS23 (1, 5 and 10 µg/µL), or DXM (10 µg/µL). **(A)** Representative biomicroscopic images of the anterior segments 24 h after LPS injection. **(B)** Clinical scores 24 h after LPS injection. The data are expressed as the mean ± SD (n = 10 eyes per group). ^##^ *p* < 0.01 vs. the control group. *p < 0.05 vs. the LPS group. **p < 0.01 vs. the LPS group.

Twenty-four hours after LPS injection, the EIU + PBS group showed severe anterior segment inflammation (clinical score 3.22 ± 0.42; Figure 1) compared with the control group (0; *p* < 0.01). The mean scores after treatment were 2.89 ± 0.31 in the 1 µg/µL KS23-treated group (*p* > 0.05 compared with the EIU + PBS group), 2.40 ± 0.50 in the 5 µg/µL KS23-treated group (*p* < 0.05 compared with the EIU + PBS group), and 1.80 ± 0.42 in the 10 µg/µL KS23-treated group (*p* < 0.01 compared with the EIU + PBS group), exhibiting clinical effects similar to those of the EIU + DXM group (clinical score 1.2 ± 0.42; *p* < 0.01 compared with the EIU + PBS group). The TQ23-treated group did not show significant differences in the clinical score compared with the EIU + PBS group.

### Effect of KS23 on Histopathological Changes

**FIGURE 2:**
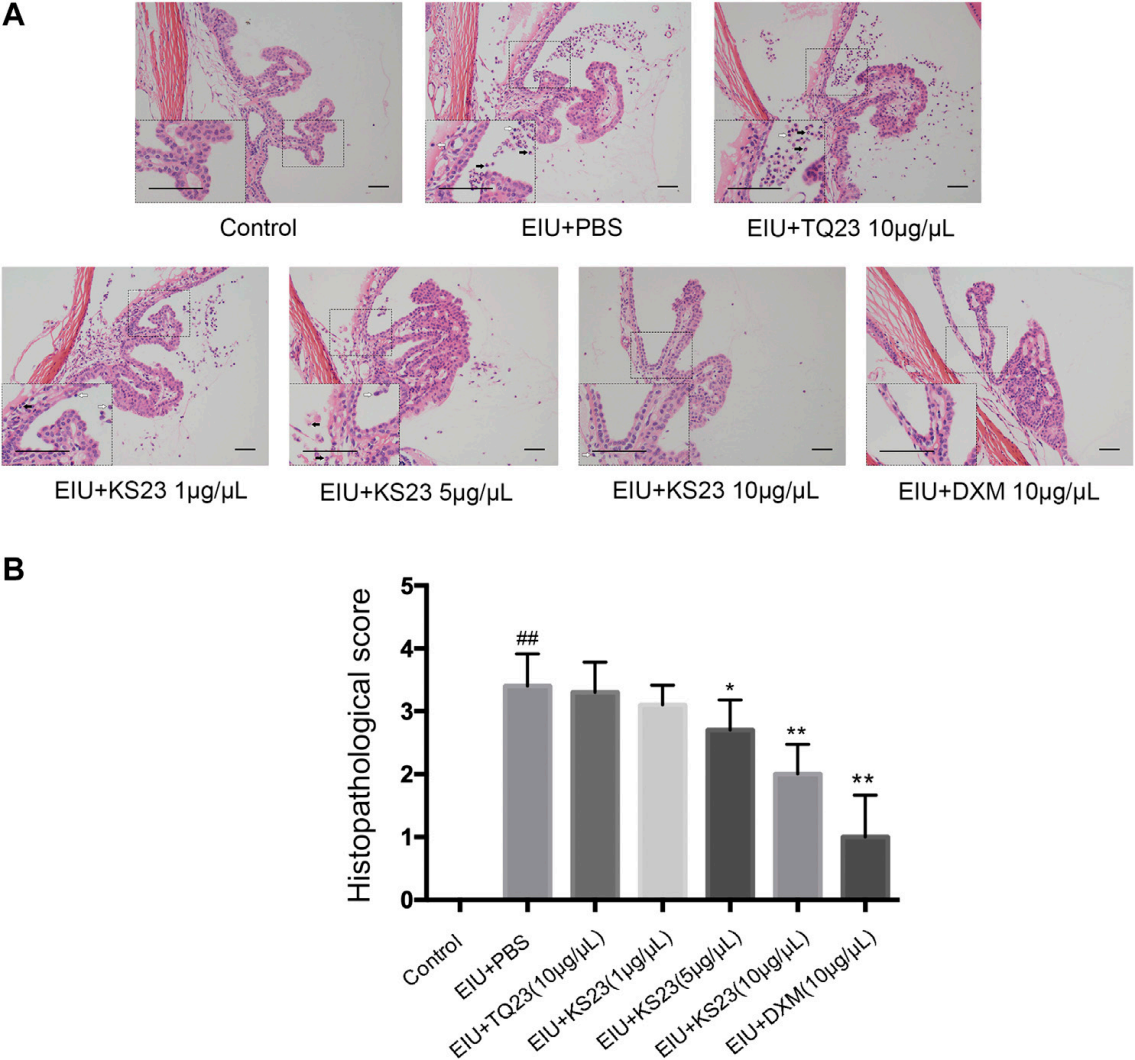
The anti-inflammatory effect of KS23 on the histopathological features of the ocular tissues of EIU rats. **(A)** H&E staining 24 h after LPS stimulation showed infiltrating inflammatory cells neutrophils (black arrow) and monocytes/macrophages (white arrow) in the iris-ciliary body region (20x, Scale bar, 50 µm). **(B)** The severity of inflammation was scored independently by two blinded experimenters. The data are expressed as the mean ± SD (n = 10 eyes per group). ^##^ *p* < 0.01 vs. the control group. *p < 0.05 vs. the LPS group. **p < 0.01 vs. the LPS group.

Consistent with clinical observations, histopathological observations showed severe cellular infiltration in the anterior chamber and ICBs in the EIU + PBS group 24 h after LPS injection (Figure 2A). The mean histopathological score significantly increased in the EIU + PBS group (3.40 ± 0.52) compared with the control group (0; *p* < 0.01). The numbers of inflammatory cells were dose-dependently decreased in the KS23-treated groups (5 µg/µL and 10 µg/µL), and the mean histopathological scores were 2.70 ± 0.35 in the 5 µg/µL KS23-treated group (*p* < 0.05 compared with the EIU + PBS group) and 2.00 ± 0.53 in the 10 µg/µL KS23-treated group (*p* < 0.01 compared with the EIU + PBS group). The EIU + TQ23 group and EIU + KS23 group (1 µg/µL) did not show significant differences in the histopathological scores compared with the EIU + PBS group (3.30 ± 0.52, 3.10 ± 0.35, respectively; all *p* > 0.05). No inflammation was observed histopathologically in the control group (Figures 2A,B).

### Effect of KS23 on Infiltrating Cells and Protein Levels in the Aqueous Humor

**FIGURE 3:**
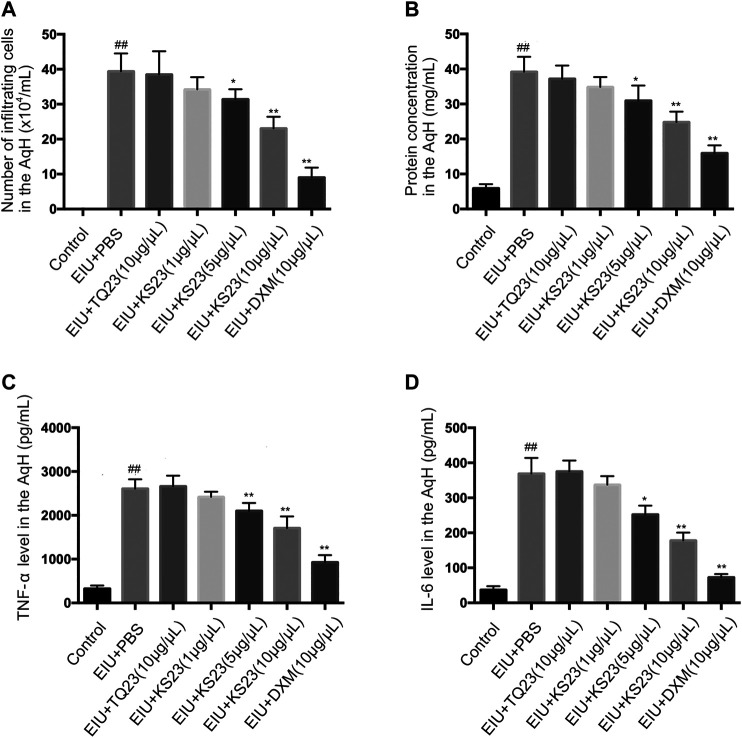
The effect of KS23 on infiltrating cells, protein concentration and pro-inflammatory cytokines in the aqueous humor. The effect of KS23 on the number of infiltrating cells **(A)**, the protein concentration **(B)**, and the concentrations of TNF-α **(C)** and IL-6 **(D)** in the aqueous humor were investigated 24 h after LPS injection. The data are expressed as the mean ± SD (n = 6 per group). ^##^ *p* < 0.01 vs. the control group. *p < 0.05 vs. the LPS group. **p < 0.01 vs. the LPS group.

To confirm the anti-inflammatory effects of KS23, we collected the AqH 24 h after LPS injection, counted the cells and measured the protein concentration. No cells were detected in the AqH of the control group, while the number of infiltrating cells was significantly increased in the EIU + PBS group (39.38 ± 4.68 × 10^4^/ml, *p* < 0.01). The KS23-treated groups (5 µg/µL and 10 µg/µL) showed significantly decreased infiltrating cells in a dose-dependent manner (31.38 ± 2.64 × 10^4^/ml and 23.04 ± 3.06 × 10^4^/ml; *p* < 0.05 and *p* < 0.01; respectively) compared to the EIU + PBS group (Figure 3A). Similarly, the protein concentration in the AqH of the EIU + PBS group (39.18 ± 3.91 mg/ml) was increased compared the control group (5.90 ± 1.12 mg/ml; *p* < 0.01), and KS23 administration (5 µg/µL and 10 µg/µL) also decreased the protein concentration in a dose-dependent manner (*p* < 0.05, *p* < 0.01, respectively) (Figure 3B). However, unlike the KS23- and DXM-treated groups, treatment with TQ23 resulted in no obvious reduction in the cell counts or protein concentration compared with the EIU + PBS group (*p* > 0.05, *p* > 0.05, respectively). These results indicate that KS23 attenuates LPS-induced cellular infiltration and reduces the protein concentration in the AqH.

### Effect of KS23 on the Tumor Necrosis Factor-α and IL-6 Levels in the Aqueous Humor

TNF-α and IL-6 are important proinflammatory cytokines that mediate the pathogenesis of uveitis (de Vos et al., 1994). We determined the levels of these cytokines in the AqH. Significantly enhanced concentrations of TNF-α and IL-6 were detected in the LPS group compared to the control group (*p* < 0.01, *p* < 0.01, respectively). Treatment with KS23 (5 and 10 µg/µL) significantly reduced TNF-α and IL-6 production compared to the EIU + PBS group. Moreover, treatment with TQ23 showed no inhibitory effects on the TNF-α and IL-6 concentrations (Figures 3C,D).

### Effect of KS23 on Cytokine Levels in Lipopolysaccharide-Stimulated RAW 264.7 Cells

**FIGURE 4:**
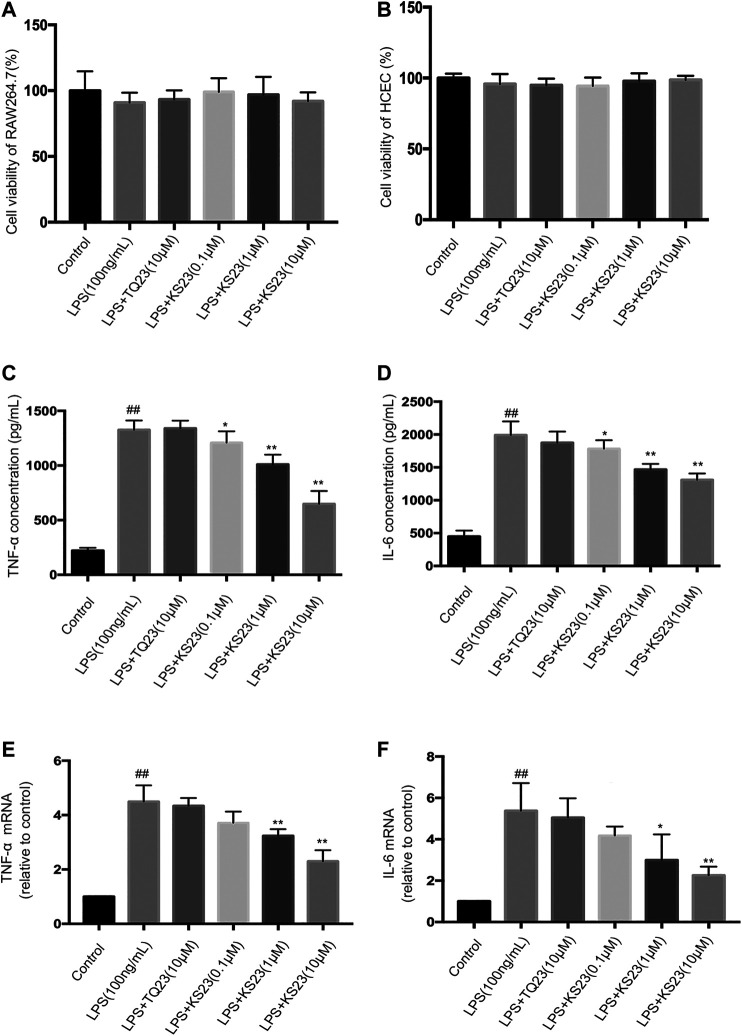
The effects of KS23 on pro-inflammatory cytokine concentrations in LPS- stimulated RAW 264.7 cells. **(A,B)** The MTS assay revealed that the KS23 and TQ23 peptides did not exhibit toxicity against RAW 264.7 cells **(A)** and HCEpiC **(B)** up to a concentration of 10 µM for 24 h **(C–F)** RAW 264.7 cells were pretreated with TQ23 (10 µM) or KS23 (0.1, 1, or 10 µM) for 30 min before being further treated with LPS (100 ng/ml) for 24 h. The concentrations of TNF-α **(B)** and IL-6 **(C)** in the culture medium were assessed by ELISA. The mRNA levels of TNF-a **(E)** and IL-6 **(F)** were assessed by qPCR analysis. GAPDH mRNA was used as the internal control. The data are expressed as the mean ± SD (n = 6 per group). ^##^ *p* < 0.01 vs. the control group. *p < 0.05 vs. the LPS group. **p < 0.01 vs. the LPS group.

The MTS assay revealed that the peptides did not exhibit toxicity in RAW 264.7 cells and primary HCEpiC up to a concentration of 10 µM (Figures 4A,B).

Therefore, to assess anti-inflammatory activity, 0.1, 1 and 10 µM KS23 was tested in the presence of LPS (100 ng/ml), as well as TQ23 at a concentration of 10 µM. The results showed that LPS stimulation significantly increased the levels of IL-6 and TNF-α, while pretreatment with KS23 significantly attenuated the production of TNF-α and IL-6 in a dose-dependent manner (Figures 4C,D), while TQ23 treatment showed no therapeutic effect. We then performed qPCR to analyze the effects of KS23 on LPS-induced TNF-a and IL-6 mRNA levels. Figures 4D,E showed that LPS treatment significantly elevated TNF-a and IL-6 mRNA expression, which was suppressed by KS23 pretreatment. However, TQ23 did not demonstrate any inhibitory effect on TNF-a and IL-6 productions. These results suggest that treatment with KS23 markedly reduced the levels of pro-inflammatory cytokines in LPS-stimulated RAW 264.7 cells, indicating the role of KS23 as an anti-inflammatory agent.

### Effect of KS23 on IκB Kinase/Inhibitor of Nuclear Factor-κB/Nuclear Factor-κB Signaling in Lipopolysaccharide-Stimulated RAW 264.7 Cells

**FIGURE 5:**
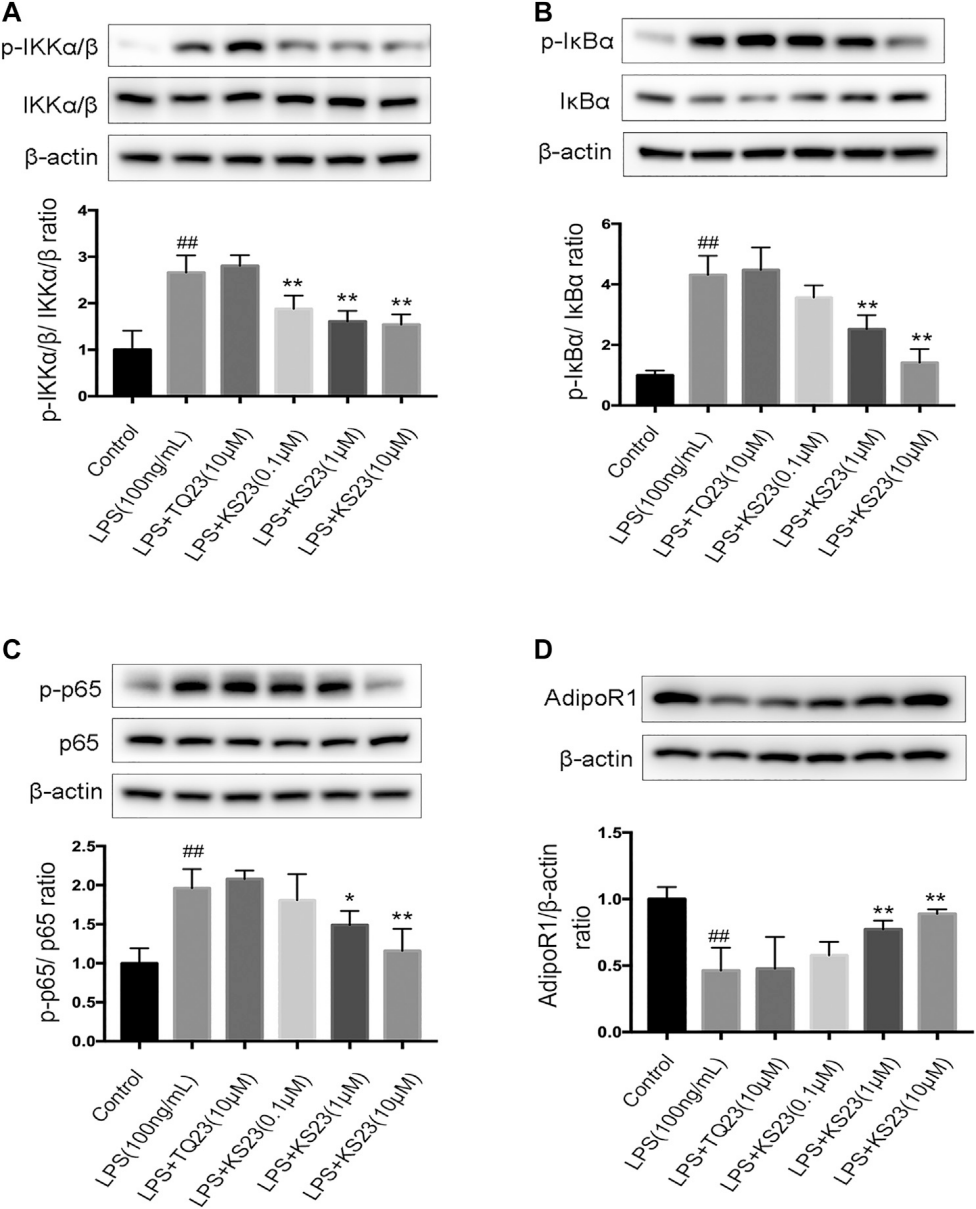
The effects of KS23 on the IKK/IκB/NF-κB pathway in LPS-stimulated RAW 264.7 cells. **(A)** RAW 264.7 cells were pretreated with PBS, TQ23 (10 µM), or KS23 (0.1, 1, or 10 µM) for 30 min before being further treated with LPS (100 ng/ml) for 1 h. The expression levels of IKKα/β, IκBα, NF-κB, phospho-IKKα/β, phospho-IκBα, and phospho-NF-κB were assessed by western blotting. β-actin was used as the internal control. The band density was determined using ImageJ software, and the relative level was calculated as the ratios of p-IKKα/β/IKKα/β **(B)**, p-IκBα/IκBα **(C)**, p-NF-κB/NF-κB **(D)**, IκBα/β-actin **(E)** and AdipoR1/β-actin **(F)**. Quantification of the band intensities after normalization to β-actin is presented. The data are expressed as the mean ± SD (n = 3 per group). ^##^ *p* < 0.01 vs. the control group. *p < 0.05 vs. the LPS group. **p < 0.01 vs. the LPS group.

NF-κB plays a pivotal role during inflammatory responses, including EIU and autoimmune uveitis (Baldwin, 1996; Kubota et al., 2009). NF-κB is a dimeric molecule composed of p50, p65 and the consensus subunit IκB. After being stimulated by endotoxin or pro-inflammatory mediators, IκB is phosphorylated by the IKK complex and degraded, and the p65 subunit then translocates into the nucleus, inducing the expression of various pro-inflammatory genes. To further characterize the molecular mechanism by which KS23 inhibits inflammatory reactions, we examined the IKK/IκB/NF-κB signaling pathway in LPS-stimulated RAW 264.7 cells. After LPS stimulation, the phosphorylation fo IKKα/β, IκBα and NF-κB all increased significantly (*p* < 0.01, *p* < 0.01, *p* < 0.01, respectively), and pretreatment with KS23 (1 and 10 µM) inhibited this phosphorylation compared with the LPS-stimulated group (Figures 5A–D). KS23 also reduced the LPS-induced degradation of the consensus subunit IκBα (Data not shown). We further investigated the effect of KS23 on AdipoR1, which is considered to be a more important receptor in the anti-inflammatory effect of adiponectin (Mandal et al., 2010; Wang et al., 2014; Jian et al., 2019). The results showed that LPS stimulation reduced the expression of AdipoR1 protein (*p* < 0.01), and KS23 treatment (1 and 10 µM) increased the expression of AdipoR1 compared to the LPS group (*p* < 0.01, *p* < 0.01, respectively), while TQ23 treatment had no effect.

### Effect of KS23 on Lipopolysaccharide-Induced Nuclear Translocation of Nuclear Factor-κB in RAW 264.7 Cells

**FIGURE 6:**
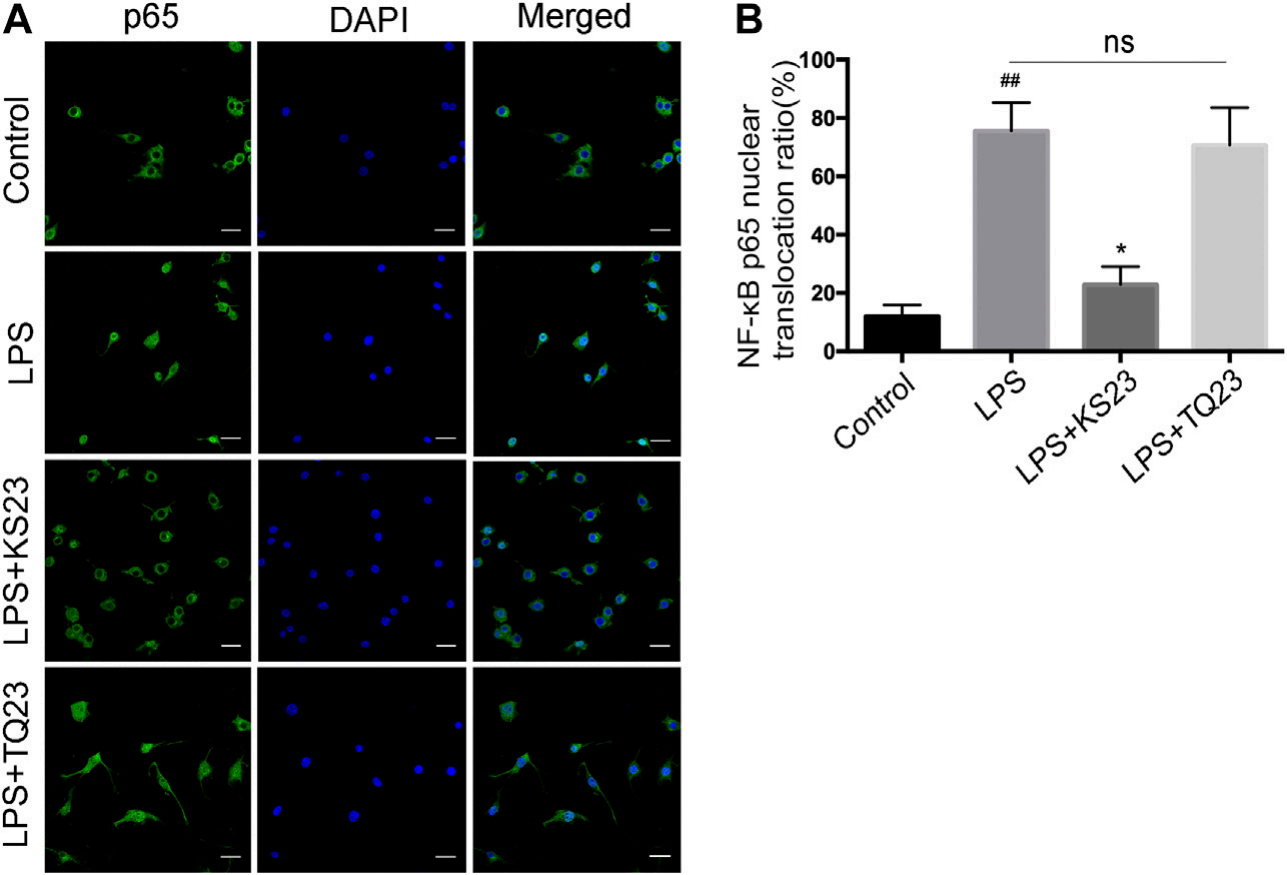
The effects of KS23 on p65 nuclear translocation in LPS-stimulated RAW 264.7 cells. **(A)** RAW 264.7 cells were pretreated with PBS, KS23 (10 µM), or TQ23 (10 µM) for 30 min before being further treated with LPS (100 ng/ml) for 1 h. Intracellular localization of the NF-κB p65 subunit was assessed with immunofluorescent staining. Scale bar: 25 µm. (Green: NF-κB p65; Blue: nucleus.) **(B)** The number of cells with nuclear translocation in six random fields was counted in a blinded fashion and is expressed as the percentage of translocated cells compared to the total number of cells. The data are expressed as the mean ± SD (n = 6 per group). ^##^ *p* < 0.01 vs. the control group. *p < 0.05 vs. the LPS group. **p < 0.01 vs. the LPS group. ns, non-significant.

To further characterize the molecular mechanism by which KS23 inhibits inflammatory reactions, we examined the translocation of NF-κB p65, which is a marker of NF-κB activation. Immunofluorescent images showed that KS23 pretreatment significantly decreased the LPS-induced nuclear translocation of NF-κB p65 from the cytosol compared to the control group (*p* < 0.05), and TQ23 did not show a similar inhibitory effect (Figures 6A,B). These results suggest that KS23 exerts its inhibitory effect by suppressing the LPS-stimulated nuclear translocation of NF-κB in RAW 264.7 macrophages.

## Disscusion

This study demonstrated the efficacy of KS23, a novel peptide derived from gAPN, in reducing the inflammatory response of EIU. KS23 improved the clinical score of EIU rats, reduced leukocyte migration and protein leakage into the AqH, and inhibited the secretion of pro-inflammatory cytokines IL-6 and TNF-α. KS23 pretreatment also inhibited the LPS-induced secretion of TNF-α and IL-6, as well as nuclear translocation of NF-κB p65 and the phosphorylation of IKKα/β/IκBα/NF-κB in RAW 264.7 cells. Together, these results indicate that inhibition of IKKα/β/IκBα/NF-κB signaling by KS23 ameliorates inflammation and suppresses the development of EIU.

Previous studies have shown that gAPN exerts anti-inflammatory effects more efficiently than full-length APN (Fruebis et al., 2001). Exogenous gAPN shows anti-inflammatory effects in various inflammatory models (Ouedraogo et al., 2007; Aprahamian et al., 2009). In eye diseases, topical application of gAPN improved the clinical symptoms of experimental dry eye syndrome and reduced ocular surface inflammation; (Li et al., 2013) however, the larger peptide fragments of adiponectin and the extreme insolubility of the C-terminal domain hinder clinical application (Otvos et al., 2011). In recent years, the use of proteins and peptides as human therapeutics has increased rapidly (Zhao et al., 2009; Niu et al., 2019). As short amino acid sequences, small molecule peptide drugs naturally mimic the binding interface between proteins (Acar et al., 2017). Therefore, we selected the KS23 sequence from gAPN for synthesis by simulation analysis and verified its efficacy in EIU and LPS-stimulated macrophage models.

EIU is a commonly used model of human acute anterior uveitis characterized by the breakdown of the blood-eye barrier, with macrophages and neutrophils leaving the iris area and infiltrating the anterior chamber, along with the induction of various cytokines and other inflammatory mediators (de Vos et al., 1994; Mérida et al., 2018). Clinical and histopathological evaluations have shown that KS23 inhibited LPS-induced blood-eye barrier destruction, manifested as protein and cell leakage into the eye. A previous study reported that infiltrated leukocytes are the main source of TNF-α and IL-6 in ocular inflammation (Crane et al., 2001).

To investigate the mechanisms responsible for these effects, we used LPS-stimulated RAW 264.7 cells as an *in vitro* model. Consistent with the *in vivo* study and previous reports, (McMenamin and Crewe, 1995; Behar-Cohen et al., 1998; Mérida et al., 2018) the levels of IL-6 and TNF-α were increased in the supernatants of murine macrophages stimulated with LPS, while KS23 prevented the production of these inflammatory markers. This result is consistent with previous reports that APN suppresses TNF-α and IL-6 production and their biological effects in adipocytes, macrophages and endothelial cells (Shibata et al., 2015). IL-6 and TNF-α are essential pro-inflammatory mediators that are increased in both the serum and AqH in animal models and patients with uveitis (Khera et al., 2010; Mesquida et al., 2017; Mérida et al., 2018). IL-6 is essential for the differentiation of neutrophils and Th17, which play a key role in autoimmune diseases by mediating tissue inflammation (Mesquida et al., 2017; Heissigerová et al., 2019). TNF-α can induce the synthesis of a variety of inflammatory cytokines, such as NF-κB, monocyte chemoattractant protein-1 (MCP-1), and intercellular adhesion molecule-1 (ICAM-1), enhance the adhesion of inflammatory cells to the endothelium, and promote the inflammatory response (Baeuerle and Henkel, 1994; Chang et al., 2010; Khera et al., 2010). The results presented here reveal that the mechanism by which KS23 inhibits EIU involves inhibition inflammatory cytokine secretion by macrophages.

To evaluate the biochemical mechanism of KS23, we further examined the NF-κB pathway. NF-κB is a critical mediator of LPS-induced inflammation (Gloire et al., 2006). NF-κB triggers various pro-inflammatory genes, including TNF-α, which also activates NF-κB, generating a positive feedback loop and leading to a cytokine cascade reaction and the amplification of inflammation (Baeuerle and Henkel, 1994; Baldwin, 1996; Bird et al., 1997). The activation of NF-κB is tightly controlled by the IKK complex and its substrate IκBα. In a previous study, we reported that KS23 can induce an anti-inflammatory response through RELA/p65 deacetylation (Niu et al., 2019). In the present study, we found that KS23 dampened the NF-κB signaling pathway by inhibiting the phosphorylation of IKKα/β and IκBα in LPS-induced RAW 264.7 cells. Because NF-κB response elements are present in the promoters of TNF-α and IL-6, the mechanism by which KS23 suppresses pro-inflammatory mediators may include blocking the IKKα/β/IκBα/NF-κB pathway.

We also measured the expression of AdipoR1, which has been shown to play an important role in the anti-inflammatory effect of adiponectin. AdipoR1 has greater gAPN binding affinity than AdipoR2, (Yamauchi et al., 2003) and the anti-inflammatory properties of full-length APN exhibit a stronger dependency upon AdipoR1 (Luo et al., 2013; Wang et al., 2014). Our previous study indicated that KS23 ameliorated inflammation in EAU by binding with AdipoR1 to activate the AMPK pathway (Niu et al., 2019). In the current study, KS23 treatment resulted in increased expression of AdipoR1, while the expression of AdipoR2 did not show significant differences (data not shown). This finding demonstrated that the anti-inflammatory effect of KS23 in EIU may be caused by KS23 binding to AdipoR1, which is consistent with the previously reported anti-inflammatory mechanisms of gAPN (Nicolas et al., 2017; Jian et al., 2019; Niu et al., 2019). Previous studies have reported that gAPN inhibits macrophage and microglial activation via the AdipoR1/NF-κB signaling pathway (Nicolas et al., 2017; Jian et al., 2019).

Collectively, the results of our study demonstrated the therapeutic effects of KS23, a novel peptide derived from gAPN, on endotoxin-induced uveitis. KS23 suppressed the infiltration of inflammatory cells and the release of inflammatory mediators, possibly by modulating the IKK/IκB/NF-κB pathway. These findings indicate that KS23 might be a potential therapeutic strategy for ocular inflammation.

## Data Availability Statement

The raw data supporting the conclusions of this article will be made available by the authors, without undue reservation, to any qualified researcher.

## Ethics Statement

The animal study was reviewed and approved by the Ethics Committee of Shanghai General Hospital, Shanghai Jiao Tong University, School of Medicine, China.

## Author Contributions

XS, SZ, TN, and KL designed the experiment. XS, HJ, XX, and YW performed the experiment. XS, TN, CW, HW and JF analyzed data and prepared the figures. XS and NT wrote the manuscript. KL conceived and supervised the study. All authors have revised the manuscript critically for important intellectual content and approved the final version to be published.

## Funding

Supported by the National Natural Science Foundation of China (81870667, 81800799), the National Key R&D Program of China (2016YFC0904800, 2019YFC0840607), the National Science and Technology Major Project of China (2017ZX09304010), the Shanghai Medical Excellent Discipline Leader Program (2017BR056), the Shanghai Municipal Education Commission–Gaofeng Clinical Medicine Grant Support Program (20161426).

## Conflict of Interest

The authors declare that the research was conducted in the absence of any commercial or financial relationships that could be construed as a potential conflict of interest.

## Abbreviations

gAPN, globular adiponectin; EIU, endotoxin-induced uveitis; LPS, lipopolysaccharide; AqH, aqueous humor; NF-κB, nuclear factor kappa B; TNF-α, tumor necrosis factor-α; IL-6, interleukin-6; ICB, iris-ciliary body; EAE, experimental autoimmune encephalomyelitis; EAU, experimental autoimmune uveitis; DXM, dexamethasone; IKK, IκB kinase; IκB, inhibitor of NF-κB; MCP-1, monocyte chemoattractant protein-1; ICAM-1, intercellular adhesion molecule-1; IL-10, interleukin-10; HO-1, heme oxygenase-1.
